# Overlapping presence of β-amyloid, tau, p-tau, and α-synuclein in skin nerve fibers in Alzheimer's disease

**DOI:** 10.1007/s00415-025-12994-5

**Published:** 2025-03-05

**Authors:** Emilie Buchholz, Marie-Luise Machule, Maria Buthut, Leon Stefanovski, Rosa Rössling, Harald Prüss

**Affiliations:** 1 German Center for Neurodegenerative Diseases (DZNE) Berlin, Berlin, Germany; 2 Department of Neurology and Experimental Neurology, Charité Universitätsmedizin Berlin, Berlin, Germany; 3 Berlin Institute of Health at Charité – Universitätsmedizin Berlin, Berlin, Germany

**Keywords:** Alzheimer’s disease, Skin biopsy, Tau, p-tau, β-amyloid, α-synuclein

## Abstract

**Objective:**

Skin nerve fiber deposition of proteins can be strongly associated with neurodegenerative diseases, such as phosphorylated α-synuclein (p-SN) in synucleinopathies. Little is known about other neurodegenerative proteins, such as tau or β-amyloid, in skin nerve fibers of patients with Alzheimer’s disease (AD) and their link to underlying neurodegeneration. We therefore aimed for describing the presence and distribution of these proteins in the skin of patients with AD and non-AD controls.

**Methods:**

Skin biopsies were taken from 45 patients with AD (*n *= 23) and non-AD controls (*n *= 22). Nerve fibers were identified using antibodies against protein gene product 9.5 (PGP9.5), and protein deposits were evaluated with double-immunostaining of β-amyloid 1-42 (Aβ1-42), p-SN, tau, and phospho-tau (p-tau).

**Results:**

Skin nerve fiber Aβ1-42 was present in 7/23 (30.4%) patients with AD and 7/22 (31.8%) controls. p-tau was detected in 12/23 (52.2%) patients with AD and 9/22 (40.9%) controls. Tau was present in 19/23 (82.6%) patients with AD and 16/22 (72.7%) controls. p-SN was detected in 12/23 (52.2%) patients with AD and 8/22 (36.4%) controls. Frequencies of deposits were not significantly different between groups and protein frequency did not correlate with severity of cognitive impairment.

**Interpretation:**

Deposits of β-amyloid 1-42, p-SN, tau, and p-tau were detected in skin nerve fibers in both patient groups; however, qualitative assessment did not discriminate between AD and non-AD patients at this sample size. Future analyses of protein distribution and spreading in peripheral nerves may give new insights into the pathophysiology of neurodegenerative diseases, but may require quantitative detection.

## Introduction

The global prevalence and incidence of patients diagnosed with Alzheimer's disease (AD) and other dementias have been steadily increasing with estimated 150 million people affected in 2050. [1] This implicates huge challenges for the health care systems including patient care, novel therapies, and improved methods for early diagnosis of neurodegenerative diseases. The current diagnostics rely on clinical criteria, brain imaging, neuropsychological assessment, and serum and CSF disease biomarkers, as tissue examination from brain biopsy is not normally available. [2] Some studies suggested that skin tissue could serve as a “window to the brain” as critical central nervous system (CNS) proteins, such as NMDA receptors or tau, are also expressed in the skin. [3, 4]

Based on the shared embryonic origin from the ectoderm, [5] neuronal and skin tissue may not only share similar protein expression, but mirror changes during degenerative diseases in the same way. Therefore, detection of proteins in the skin could represent an easily accessible peripheral biomarker of neurodegenerative diseases. In synucleinopathies, detection of skin phosphorylated α-synuclein (p-SN) is a promising new approach for diagnosis and differentiation from other neurodegenerative diseases and controls. [6, 7]

For other proteinopathies, such as tauopathies including AD, much less is known on tau and β-amyloid (Aβ) expression in the skin with inconsistent ability to discriminate between patients and controls. [8, 9] In this pilot study, we therefore aimed to investigate the distribution and staining patterns of different neurodegenerative proteins in the skin of patients with and without AD. We hypothesized that colocalization of tau and Aβ1–42 in nerve fibers is more common in patients with AD.

## Methods

### Patients

In total, 45 subjects were recruited from the memory clinic of the Department of Neurology at Charité Universitätsmedizin Berlin, Campus Mitte. This study was approved by the Charité Ethics committee (number EA4/221/20) and all patients gave written informed consent prior to study inclusion. Samples were handled with pseudonymized identifiers and investigators were blinded to the diagnosis.

Patients were diagnosed according to current clinical guidelines. [10] Severity of dementia was rated with the mini-mental state examination (MMSE) and classified as mild (MMSE 21–26), moderate (MMSE 10–20), or severe (MMSE <10). [11] The patients were grouped as follows: *(1)* patients with Alzheimer’s disease (*n* = 23) and *(2)* patients without AD (*n* = 22) including patients with affective disorders (*n* = 7), Parkinson’s disease (*n* = 2), Lewy body dementia (*n* = 2), frontotemporal dementia (*n* = 1), corticobasal syndrome (*n* = 1), CLIPPERS (*n* = 1), migraine (*n* = 1), myasthenia gravis (*n* = 1), dystonic tremor (*n* = 1), IgLON5 disease (*n* = 1), NMDAR encephalitis/MS overlap (*n* = 1), epilepsy (*n* = 1), cervical dystonia (*n* = 1), and stroke (*n* = 1).

### Skin biopsy and fixation

All patients underwent a 3 mm skin punch biopsy after local anesthesia with 2% lidocaine from the lateral malleolus area, ~10 cm above the ankle. The sample was placed in Zambonis solution at 4 °C overnight, washed in 1x PBS and then placed in cryoprotectant solution (glycerol, Sörensen buffer, and water) at 4 °C overnight. Then, the samples were frozen and stored at −80 °C. The whole biopsy was cut into 50 µm sections using a cryostat. The sections that were used for the staining were immediately placed in 1x PBS.

### Immunofluorescence staining and controls

The free-floating skin sections were double-immunostained using commercial antibodies. To visualize peripheral nerve fibers, two PGP9.5 antibodies were used (ab72911, monoclonal, mouse, Abcam, 1:500 and ab108986, monoclonal, rabbit, Abcam, 1:500). To investigate potential protein colocalization, antibodies against Aβ1–42 (bs-0107R, polyclonal, rabbit, Bioss Antibodies, 1:500), p-tau (AT8) (MN1020, mouse, monoclonal, Invitrogen, 1:500), tau (Ser262) (ab64193, rabbit, polyclonal, Abcam, 1:500), and p-SN (ab51253, rabbit, monoclonal, Abcam, 1:500) were used. As positive controls, brain tissue from the superior temporal gyrus of a patient with AD and skin tissue of two patients with PD were immunostained. Negative controls were brain tissue sections of a patient with amyotrophic lateral sclerosis (ALS). For the systematic staining, four sections per antibody plus one control using secondary only were stained for each patient. The sections were blocked for one hour at room temperature and then incubated in the primary antibody dilutions overnight on a shaker at 4 °C. The next day, the sections were washed in 1x PBS and incubated light protected on a shaker in the secondary dilutions for two hours at room temperature. After the incubation, the sections were washed in 1x PBS and mounted on slides.

### Confocal microscopy and evaluation

Each skin section was screened for immunopositive signal in the epidermis, sweat glands, arrector pili muscles (MAP), and hair follicles using confocal microscopy (Nikon A1Rsi+ laser-scanning confocal microscope). Single frame z-stack images were analyzed with consecutive 2 µm steps. A patient was counted as positive, if at least one nerve fiber had a positive co-staining. Criteria for a positive result were a completely co-localized strong signal and clear morphology within the fiber tracking through at least two z-planes. Any areas of tissue damage as well as dot like artifacts were excluded and not considered as positive, as previously described. [12]

### Statistical analysis

Statistical analyses were performed using SPSS version 30.0 (IBM Corp) software for Windows. For the analysis of categorical variables, the 2-sided Fisher–Freeman–Halton exact test was performed. A significance level of *p* <0.05 was assumed.

## Results

**Fig. 1:**
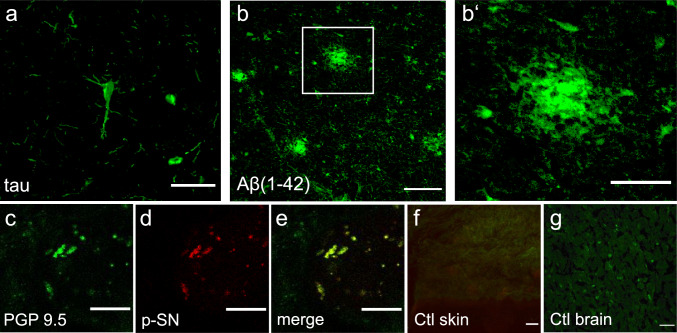
Antibody staining patterns in brain and skin tissue, **a,b** The gyrus temporalis superior of brain sections from a patient with Alzheimer‘s disease serves as positive controls for the immunofluorescence staining of neurons with intracellular tau tangles (**a**) and extracellular Aβ1–42 plaques (**b**, enlarged in **b**‘). **c-e** Skin biopsy sections of a patient with Parkinson‘s disease shows PGP9.5-positive nerve fibers (**c**) colocalizing with abnormal neuronal p-SN (**d**, merged in **e**). **f** Negative control staining of the skin using the secondary antibody only. **g** In a disease control patient with amyotrophic lateral sclerosis, there was no Aβ1–42 plaque staining on brain sections. Scale bars indicate 100 µm in (**a–b**) and (**f–g**), and 20 µm in (**c–e**)

We enrolled 45 patients with a mean age of 68.5 years (SD±10). The general characteristics of the populations are presented in Table 1. Patients were categorized into two groups: patients with AD (*n* = 23) and control patients without AD (*n* = 22). Stainings were validated using positive control tissue showing the typical neuropathological characteristics, i.e., intracellular tau (Fig. 1a and extracellular β-amyloid (Fig. 1b-b’) in brain sections of an AD patient, and p-SN in the skin nerve fibers of a PD patient (Fig. 1c-e). Negative controls consisted of omission of the secondary antibody in skin sections (Fig. 1f) and absence of β-amyloid in ALS brain tissue (Fig. 1g).

**Table 1.** General patient characteristics and frequencies of deposition of neurodegenerative proteins in skin nerve fibers and epidermis

	Sex (f/m)	Age (mean ±SD)	Disease duration (years± SD)	Aβ1–42	p-tau	tau	p-SN	p-tau	p-SN	p-Tau	p-SN
				Skin nerve fibers				Epidermal staining (cytoplasmic)		Epidermal staining (nuclear)	
AD (n=23)	7/16	69.5 ±7.1	2.9 ±1.6	7/23 (30.4%)	12/23 (52.2%)	19/23 (82.6%)	12/23 (52.2%)	5/23 (21.7%)	12/23 (52.2%)	10/23 (43.5%)	15/23 (65.2%)
Non-AD (n=22)	9/13	67.4 ±12.4	6.7 ±7.8	7/22 (31.8%)	9/22 (40.9%)	16/22 (72.7%)	8/22 (36.4%)	2/22 (9.1%)	9/22 (40.9%)	12/22 (54.6%)	11/22 (50%)

**Fig. 2:**
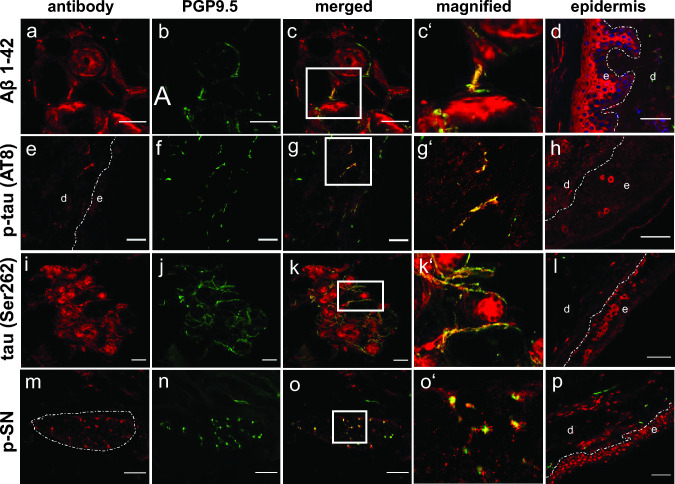
Deposits of neurodegeneration-associated proteins in skin nerve fibers and epidermis in patients with neurodegenerative diseases, **a-c**’ Aβ1–42 deposits (**a**) were detected in PGP9.5-positive nerve fibers (**b**, merged in **c** with higher-resolution insert in **c**’) around a sweat gland in skin biopsy sections, exemplarily shown in a patient with Lewy body dementia. **d** Cytoplasmic staining of Aβ1–42 in keratinocytes in a patient with Alzheimer’s disease (blue, DAPI-positive cell nuclei). **e-g’** p-tau (AT8) (**e**) in subepidermal nerve fibers (**f**) of a patient with Alzheimer’s disease. **h** p-tau (AT8) also stained the cytoplasm of keratinocytes in an Alzheimer’s disease patient**. i-k’** Tau deposits (**i**) in PGP9.5-positive nerve fibers (**j**) of a sweat gland in a patient with Alzheimer’s disease. **l** Tau-positive immunofluorescence in the cytoplasm of keratinocytes in a patient with affective disorder. **m-o’** p-SN staining (**m**) colocalizing with nerve fibers (**n**) in an arrector pili muscle (**m**, dotted line) of a patient with Parkinson’s disease. **p** Nuclear staining pattern of p-SN in keratinocytes of a patient with Alzheimer’s disease., *e*, epidermis; *d*, dermis; dotted line (**d, h, l, p**) indicates epidermal–dermal junction. Scale bars indicate 50 µm

In the systematic skin stainings of all patients, the four commercial antibodies labeled peripheral nerve fibers with different frequencies, as detailed below and summarized in Table 1. We frequently found overlapping presence of different proteins in the same patient with 14/45 for two antibodies, 12/45 for three antibodies, and 3/45 for all four tested antibodies. Interestingly, staining of keratinocytes of the epidermal layer was frequently seen (Fig. 2). We frequently found dot like artifacts in the lumen of sweat glands across all groups (not shown).

### β-Amyloid 1–42

Aβ1–42 deposits were detected in PGP9.5-positive nerve fibers in 14 of 45 subjects in both groups, with 7/23 (30.4%) patients in the AD group and 7/22 (31.8%) in the control group (Table 1). There were no significant differences between the groups. Frequency of deposits did not correlate with MMSE values (not shown). Aβ1–42 deposits were predominantly localized in sweat glands (12/14) (Fig. 2a-c’) but also in the subepidermal region (6/14) and arrector pili muscles (MAP) (2/14).

Homogeneous cytoplasmic staining for Aβ1–42 was seen in the epidermal layer of all patients (Fig. 2d) without differences between the groups. Likewise, immunopositivity was detected in the cytoplasm of skin fibroblasts and in the epithelium of skin appendages (not shown).

### p-tau (AT8)

P-tau was observed in PGP9.5-positive nerve fibers in 20 of 45 subjects, with 12/23 (52.2%) patients in the AD group and 9/22 (40.9%) in the control group (Table 1). There were no significant differences between the groups. Frequency of deposits did not correlate with MMSE values (not shown). Immunopositive signals were found predominantly in the subepidermal skin layers (16/20) (Fig. 2e-g’) followed by sweat glands (9/20) and MAP (3/20).

In the epidermal layer, p-tau staining showed either a cytoplasmic (Fig. 2h) or nuclear staining pattern of the keratinocytes. Cytoplasmic patterns were observed in 5/23 (21.7%) in the AD group, 2/22 (9.1%) of the controls (Table 1). A nuclear pattern was observed in 10/23 (43.5%) patients in the AD group and 12/22 (54.6%) of the controls. Nuclear staining patterns were also found in fibroblasts or around blood vessels (not shown).

### tau (Ser262)

Tau immunopositivity in peripheral nerve fibers was detected in 35/45 patients with 19/23 (82.6%) patients in the AD group and 16/22 (72.7%) of the controls (Table 1). There were no significant differences between the groups. Frequency of deposits did not correlate with MMSE values (not shown). Overlap with PGP9.5 staining was predominantly found in sweat glands (27/35) (Fig. 2i-k’) followed by subepidermal layer (16/35), MAP (4/35), and in one patient in nerve fibers surrounding the hair follicle (not shown).

All patients showed either a homogeneous cytoplasmic epidermal staining or the cytoplasm of single keratinocytes (Fig. 2l) with no differences between the groups.

### p-SN

p-SN deposits were detected in PGP9.5-positive nerve fibers in 20 of 45 subjects in both groups, with immunopositivity in 12/23 (52.2%) of the patients in the AD group and 8/22 (36.4%) in the control group (Table 1). There were no statistical differences between groups. Frequency of deposits did not correlate with MMSE values (not shown). Co-staining with PGP9.5-positive fibers was predominantly found in sweat glands (15/20) followed by nerve fibers of the MAP (9/20) (Fig. 2m-o’), subepidermal layer (4/20), and in one patient in fibers of the hair follicle.

Keratinocytes were often immunopositive following a nuclear (Fig. 2p) or cytoplasmic staining pattern. Nuclear patterns were observed in 15/23 (65.2%) patients in the AD group and 11/22 (50%) in the control group. Cytoplasmic patterns were observed in 12/23 (52.2%) patients in the AD group and 9/22 (40.9%) of the controls.

## Discussion

The neurodegenerative disease-associated proteins Aβ1–42, p-tau (AT8), tau (Ser262), and p-SN were detected in skin nerve fibers and keratinocytes of patients with AD and non-AD controls. However, frequency and distribution of all four proteins were not significantly different between both groups and no correlation with severity of cognitive impairment evaluated by MMSE was found. To our knowledge, deposition of Aβ1–42 has not been described before in skin nerve fibers, compared to the other neurodegenerative disease-associated proteins.

Previous work on skin deposition of Aβ, p-tau, and tau resulted in inconsistent findings, potentially related to a number of different staining methods and antibodies. For example, Aβ was detected in skin nerve fibers and sweat glands of patients with AD and controls in one study, but without group differences. [13] Other work did not specify localization in nerve fibers, but detected Aβ deposits in the endothelium of dermal blood vessels and around the basement membrane of patients with AD, vascular dementia, and elderly controls, but not younger controls. [9, 14] The amyloid precursor protein (APP) was not observed in the skin of AD patients and controls. [9, 15]

Only a few studies investigated tau and p-tau deposits in skin biopsies. While three studies detected tau (but not p-tau) using immunofluorescence (IF) in skin samples of patients with tauopathies and controls, [4, 16, 17], another study could not find tau in the skin of AD patients and controls. [18] The presence of tau along nerve fibers was demonstrated in patients with PD, multiple system atrophy (MSA), progressive supranuclear palsy (PSP), and corticobasal syndrome (CBD) as well as healthy controls using IF, while only tau quantification by ELISA was able to discriminate PSP/CBD as tauopathies from the other disease entities. The same study could not demonstrate p-tau using IF but with Western blot in patients with neurodegenerative disorders and healthy controls. [17] In contrast, p-tau (AT8) IF deposits were significantly more common in the nuclei of keratinocytes of AD patients compared to healthy and non-neurodegenerative controls. The same study demonstrated cytoplasmic staining of keratinocytes using an antibody against p-tau (PHF) with no significant difference between groups. [8] AT8 positivity was also demonstrated in patients with PSP with significantly higher frequencies compared to healthy controls. [19]

In a recent study, p-SN was detected in 95.5% of 277 patients with synucleinopathies including PD, MSA, dementia with Lewy bodies (DLB), and pure autonomic failure (PAF), but only in 3.3% of 151 controls showing a high sensitivity and specificity. [7] Several studies demonstrated p-SN in CSF and p-SN deposits in peripheral tissues including colon, submandibular gland, and skin. [20]

Our study demonstrated the common presence of several neurodegeneration-associated proteins in the skin of the same patients and across different disease groups, in line with previous findings. For example, PD patients showed immunopositivity to AT8, which was more frequent compared to healthy controls. [19] In another study, p-SN skin deposits were detected in 7.7% of patients with the tauopathies PSP and CBD. [21] Likewise in the brain, tau was present in ~50% of patients with PD and pathological synuclein in ~31.5–60.7% of patients with AD, [22–24] suggesting similar overlapping patterns across compartments. This could explain higher levels of p-SN in our non-synucleinopathy patients. To our knowledge, we demonstrated the presence of p-SN in the skin nerve fibers of AD patients for the first time.

This study has several limitations. At the clinical level, the sample size was relatively small and the non-AD control group was heterogeneous and did not include healthy controls. In particular, AD pathology may have co-occurred in some non-AD subjects. This may have had an impact on the presence of neurodegeneration-associated proteins. For obvious reasons, diagnoses were clinical and no autopsy confirmation available. Another factor of bias may be the biopsy site. For example in the skin, p-SN follows a proximal-distal gradient with higher frequencies in the posterior cervical spine compared to thoracic spine regions and even lower frequency in the lateral malleolus area of the leg. In PD patients, 93% had p-SN-positive skin biopsies of the posterior cervical spine area, but only 17% in biopsies of the distal leg. [25, 26] Thus, using biopsies of the lateral malleolus area in our study might have influenced the results, and a biopsy from the cervical spine area would have allowed evaluation of a possible gradient for the presence of tau, p-tau, and Aβ1–42.

As qualitative methods such as IF for tau detection did not discriminate between different diseases, quantitative methods such as ELISA might be required. [17] Along these lines, novel *in vivo* methods like seeding amplification assays from skin including Real-Time Quaking-Induced Conversion (RT-QuIC) are promising new tools, for example, in α-synucleinopathies [27] and tauopathies. [28, 29] These methods might likewise demonstrate aggregation and therefore probable pathogenicity which could help explain peripheral symptoms of neurodegenerative diseases. The distribution of neurodegenerative disease-associated proteins in sweat glands, MAP, or hair follicles may influence autonomic dysregulations, [30] and the presence of these proteins in keratinocytes could potentially affect intraepidermal nerve fiber loss and therefore the development of peripheral sensory neuropathies.

Taken together, detection of a spectrum of neurodegenerative disease-associated proteins is possible using skin biopsies, supporting clinical diagnoses of neurodegenerative diseases. With further research, skin biopsies may become helpful tools for understanding disease pathology including the peripheral distribution and the multi-step contribution to the development of neurodegenerative diseases.

## Data Availability

The data that support the findings of this study are available from the corresponding author upon reasonable request.

## References

[1] Nichols E, Steinmetz JD, Vollset SE et al (2022) Estimation of the global prevalence of dementia in 2019 and forecasted prevalence in 2050: an analysis for the global burden of disease study 2019. Lancet Public Health 7:e105–e125. 10.1016/S2468-2667(21)00249-8 34998485 10.1016/S2468-2667(21)00249-8 PMC8810394

[2] Elahi FM, Miller BL (2017) A clinicopathological approach to the diagnosis of dementia. Nat Rev Neurol 13:457–476. 10.1038/nrneurol.2017.96 28708131 10.1038/nrneurol.2017.96 PMC5771416

[3] Nahm WK, Philpot BD, Adams MM et al (2004) Significance of N-methyl-D-aspartate (NMDA) receptor-mediated signaling in human keratinocytes. J Cell Physiol 200:309–317. 10.1002/jcp.20010 15174101 10.1002/jcp.20010

[4] Dugger BN, Whiteside CM, Maarouf CL et al (2016) The presence of select tau species in human peripheral tissues and their relation to Alzheimer’s disease. J Alzheimers Dis 51:345–356. 10.3233/JAD-150859 26890756 10.3233/JAD-150859 PMC6074044

[5] Jameson C, Boulton KA, Silove N et al (2023) Ectodermal origins of the skin-brain axis: a novel model for the developing brain, inflammation, and neurodevelopmental conditions. Mol Psychiatry 28:108–117. 10.1038/s41380-022-01829-8 36284159 10.1038/s41380-022-01829-8 PMC9812765

[6] Donadio V, Incensi A, Rizzo G et al (2020) Skin biopsy may help to distinguish multiple system atrophy-parkinsonism from parkinson’s disease with orthostatic hypotension. Mov Disord 35:1649–1657. 10.1002/mds.28126 32557839 10.1002/mds.28126

[7] Gibbons CH, Levine T, Adler C et al (2024) Skin biopsy detection of phosphorylated α-synuclein in patients with synucleinopathies. JAMA 331:1298–1306. 10.1001/jama.2024.0792 38506839 10.1001/jama.2024.0792 PMC10955354

[8] Rodríguez-Leyva I, Chi-Ahumada E, Calderón-Garcidueñas AL et al (2015) Presence of phosphorylated tau protein in the skin of Alzheimer´s disease patients. J Mol Biomark Diagn. 10.4172/2155-9929.S6-005

[9] Heinonen O, Soininen H, Syrjänen S et al (1994) beta-amyloid protein immunoreactivity in skin is not a reliable marker of Alzheimer’s disease: an autopsy-controlled study. Arch Neurol 51:799–804. 10.1001/archneur.1994.00540200075019 8042928 10.1001/archneur.1994.00540200075019

[10] Jack CR, Andrews JS, Beach TG et al (2024) Revised criteria for diagnosis and staging of Alzheimer’s disease: Alzheimer’s association workgroup. Alzheimers Dement 20:5143–5169. 10.1002/alz.13859 38934362 10.1002/alz.13859 PMC11350039

[11] (2018) Dementia (National Institute for Health and Care Excellence (Great Britain)). National Institute for Health and Care Excellence, London

[12] Wang N, Garcia J, Freeman R et al (2020) Phosphorylated alpha-synuclein within cutaneous autonomic nerves of patients with parkinson’s disease: the implications of sample thickness on results. J Histochem Cytochem 68:669–678. 10.1369/0022155420960250 32921251 10.1369/0022155420960250 PMC7534099

[13] Ikeda M, Shoji M, Yamaguchi H et al (1993) Diagnostic significance of skin immunolabelling with antibody against native cerebral amyloid in Alzheimer’s disease. Neurosci Lett 150:159–161. 10.1016/0304-3940(93)90525-p 8469414 10.1016/0304-3940(93)90525-p

[14] Joachim CL, Mori H, Selkoe DJ (1989) Amyloid beta-protein deposition in tissues other than brain in Alzheimer’s disease. Nature 341:226–230. 10.1038/341226a0 2528696 10.1038/341226a0

[15] Arai H, Lee VM, Messinger ML et al (1991) Expression patterns of beta-amyloid precursor protein (beta-APP) in neural and nonneural human tissues from Alzheimer’s disease and control subjects. Ann Neurol 30:686–693. 10.1002/ana.410300509 1763893 10.1002/ana.410300509

[16] Dugger BN, Hoffman BR, Scroggins A et al (2019) Tau immunoreactivity in peripheral tissues of human aging and select tauopathies. Neurosci Lett 696:132–139. 10.1016/j.neulet.2018.12.031 30579993 10.1016/j.neulet.2018.12.031 PMC7357994

[17] Vacchi E, Lazzarini E, Pinton S et al (2022) Tau protein quantification in skin biopsies differentiates tauopathies from alpha-synucleinopathies. Brain 145:2755–2768. 10.1093/brain/awac161 35485527 10.1093/brain/awac161

[18] Miklossy J, Taddei K, Martins R et al (1999) Alzheimer disease: curly fibers and tangles in organs other than brain. J Neuropathol Exp Neurol 58:803–814. 10.1097/00005072-199908000-00003 10446805 10.1097/00005072-199908000-00003

[19] Rodríguez-Leyva I, Chi-Ahumada EG, Carrizales J et al (2016) Parkinson disease and progressive supranuclear palsy: protein expression in skin. Ann Clin Transl Neurol 3:191–199. 10.1002/acn3.285 27042679 10.1002/acn3.285 PMC4774258

[20] Chahine LM, Beach TG, Brumm MC et al (2020) In vivo distribution of α-synuclein in multiple tissues and biofluids in parkinson disease. Neurology 95:e1267–e1284. 10.1212/WNL.0000000000010404 32747521 10.1212/WNL.0000000000010404 PMC7538226

[21] Giannoccaro MP, Avoni P, Rizzo G et al (2022) Presence of skin α-synuclein deposits discriminates parkinson’s disease from progressive supranuclear palsy and corticobasal syndrome. J Parkinsons Dis 12:585–591. 10.3233/JPD-212904 34864689 10.3233/JPD-212904 PMC8925116

[22] van der Gaag BL, Deshayes NAC, Breve JJP et al (2024) Distinct tau and alpha-synuclein molecular signatures in Alzheimer’s disease with and without lewy bodies and parkinson’s disease with dementia. Acta Neuropathol 147:14. 10.1007/s00401-023-02657-y 38198008 10.1007/s00401-023-02657-y PMC10781859

[23] Mikolaenko I, Pletnikova O, Kawas CH et al (2005) Alpha-synuclein lesions in normal aging, parkinson disease, and Alzheimer disease: evidence from the baltimore longitudinal study of aging (BLSA). J Neuropathol Exp Neurol 64:156–162. 10.1093/jnen/64.2.156 15751230 10.1093/jnen/64.2.156

[24] Hamilton RL (2000) Lewy bodies in Alzheimer’s disease: a neuropathological review of 145 cases using alpha-synuclein immunohistochemistry. Brain Pathol 10:378–384. 10.1111/j.1750-3639.2000.tb00269.x 10885656 10.1111/j.1750-3639.2000.tb00269.x PMC8098522

[25] Donadio V, Incensi A, Rizzo G et al (2017) Spine topographical distribution of skin α-synuclein deposits in idiopathic parkinson disease. J Neuropathol Exp Neurol 76:384–389. 10.1093/jnen/nlx021 28402459 10.1093/jnen/nlx021

[26] Gibbons C, Wang N, Rajan S et al (2023) Cutaneous α-synuclein signatures in patients with multiple system atrophy and parkinson disease. Neurology 100:e1529–e1539. 10.1212/WNL.0000000000206772 36657992 10.1212/WNL.0000000000206772 PMC10103107

[27] Yoo D, Bang J-I, Ahn C et al (2022) Diagnostic value of α-synuclein seeding amplification assays in α-synucleinopathies: a systematic review and meta-analysis. Parkinsonism Relat Disord 104:99–109. 10.1016/j.parkreldis.2022.10.007 36289019 10.1016/j.parkreldis.2022.10.007

[28] Dellarole IL, Vacchi E, Ruiz-Barrio I et al (2024) Tau seeding activity in skin biopsy differentiates tauopathies from synucleinopathies. NPJ Parkinsons Dis 10:116. 10.1038/s41531-024-00728-9 38879633 10.1038/s41531-024-00728-9 PMC11180195

[29] Wang Z, Wu L, Gerasimenko M et al (2024) Seeding activity of skin misfolded tau as a biomarker for tauopathies. Mol Neurodegener 19:92. 10.1186/s13024-024-00781-1 39609917 10.1186/s13024-024-00781-1 PMC11606191

[30] Wang N, Gibbons CH, Lafo J et al (2013) α-Synuclein in cutaneous autonomic nerves. Neurology 81:1604–1610. 10.1212/WNL.0b013e3182a9f449 24089386 10.1212/WNL.0b013e3182a9f449 PMC3806913

